# Association of Race and Major Adverse Cardiac Events (MACE): The Atherosclerosis Risk in Communities (ARIC) Cohort

**DOI:** 10.1155/2020/7417242

**Published:** 2020-03-21

**Authors:** Ericha G Franey, Donna Kritz-Silverstein, Erin L Richard, John E Alcaraz, Caroline M Nievergelt, Richard A Shaffer, Vibha Bhatnagar

**Affiliations:** ^1^Graduate School of Public Health, San Diego State University, San Diego, CA, USA; ^2^Department of Family Medicine and Public Health, University of California San Diego, La Jolla, California, USA; ^3^Department of Psychiatry, University of California San Diego, La Jolla, California, USA

## Abstract

**Background and Aims:**

To evaluate the association of self-reported race with major adverse cardiac events (MACE) and modification of this association by paraoxonase gene (*PON*1, *PON*2, and *PON*3) single nucleotide polymorphisms (SNPs).

**Methods:**

Included in this longitudinal study were 12,770 black or white participants from the Atherosclerosis Risk in Communities (ARIC) cohort who completed a baseline visit (1987–1989) with *PON* genotyping. Demographic, behavioral, and health information was obtained at baseline. MACE was defined as first occurrence of myocardial infarction, stroke, or CHD-related death through 2004. Cox proportional hazards regression was used to evaluate the association between race and MACE after adjustment for age, gender, and other demographic and cardiovascular risk factors such as diabetes and hypertension. Modification of the association between *PON* SNPs and MACE was also assessed.

**Results:**

Blacks comprised 24.6% of the ARIC cohort; overall, 14.0% of participants developed MACE. Compared with whites, blacks had 1.24 times greater hazard of MACE (OR = 1.24,95%CI = 1.10,1.39) than whites after adjusting for age, gender, BMI, cigarette and alcohol use, educational and marital status, and aspirin use. This association became nonsignificant after further adjustment for high cholesterol, diabetes, and hypertension. None of the evaluated SNPs met the significance level (*p* < 0.001) after Bonferroni correction for multiple comparisons.

**Conclusions:**

No association between race and MACE was identified after adjusting for high cholesterol, diabetes, and hypertension, suggesting that comorbidities are major determinants of MACE; medical intervention with focus on lifestyle and health management could ameliorate the development of MACE. Further studies are needed to confirm this observation.

## 1. Introduction

Cardiovascular disease (CVD) is the leading cause of death in most racial and ethnic groups in the United States including blacks and whites, accounting for 1 in 4 deaths. [1] Composite cardiovascular outcomes create an endpoint which includes first occurrence of prespecified cardiac events, commonly referred to as MACE (major adverse cardiac events), increasing statistical power and improving detectability of clinically meaningful differences. [2].

Prior studies have found racial and ethnic differences in MACE. For example, in a cohort of 864 renal transplant patients where MACE was defined as a composite of nonfatal myocardial infarction, coronary intervention and cardiac death, Prasad et al. found a higher rate of MACE after transplant for South Asians compared to East Asians, whites and blacks (4.4/100 patient-years vs. 1.61, 1.31, and 1.16/100 patient-years, respectively). [3] Napan et al. in a cohort of 1,438 patients undergoing percutaneous coronary intervention, found 52% greater hazard (HR = 1.52; 95%CI = 1.18, 1.96) of MACE (composite of death, myocardial infarction, and revascularization) among blacks compared to non-blacks after adjusting for age, gender, CVD risk factors, socioeconomic status, and other potential confounders. [4].

The association between race and MACE may be modified by genetics. Paraoxonase (*PON*) genes play an important role in the translation of enzymes that inhibit the arterial formation of cholesterol causing atherosclerosis leading to development of CVD. [5] However, results of the few studies examining the association between *PON* single nucleotide polymorphisms (SNPs) and MACE have been inconsistent. For instance, Kang et al. reported a marginal effect of the *PON1* Q192R SNP on risk of MACE (*p* < 0.05) in a case-control study of 538 Han Chinese men and women aged 26–80 years who were undergoing percutaneous coronary intervention and followed up to 1 year for MACE. [6] A prospective US cohort study of 1399 men and women aged 55 years and older who were undergoing coronary angiography found that participants with the lowest *PON1* activity had 3.4 times greater hazard of MACE (myocardial infarction, stroke or death) compared to those with the highest levels (HR = 3.4; 95%CI = 2.0, 5.9) [7]. To our knowledge, no prior study has examined the *PON* gene effect on the association between race and MACE.

The purpose of this study was to evaluate the association of black and white race with MACE and to evaluate the effect of paraoxonase single nucleotide polymorphisms (SNPs) on this association using data from a large cohort of older men and women (ARIC).

## 2. Material and Methods

This study was approved by the University of California San Diego Human Research Protections Program (#160359X); data were collected through authorized access from dbGaP. The ARIC [8] research study was supported by the National Heart, Lung and Blood Institute (NHLBI) of the National Institutes of Health; each site obtained institutional review board approval and written informed consent from study participants. Data analyses performed using SAS® University Edition (SAS Institute, Cary, NC).

### 2.1. ARIC

The Atherosclerosis Risk in Communities Study (ARIC) [8] is a prospective cohort study enrolling approximately 4,000 participants selected by probability sampling at each of four communities in the United States (Washington County, MD; Forsyth County, NC; Jackson, MS; and Minneapolis, MN). A total of 15,972 study participants were examined at baseline (1987–1989) and at four follow-up visits conducted at three-year intervals through 2013. Since 2012, participants have been contacted annually by telephone to assess health status. Enrolled study participants were black (27%) and white (73%) men and women, aged 45–64 years. Study objectives were to investigate the etiology and natural history of atherosclerosis including the risk factors and progression of subclinical to clinical cardiovascular disease events.

### 2.2. Participants

This study was limited to the 12,770 ARIC participants (24.6% black, 75.4% white) for whom *PON* genotyping data was available who also experienced a first occurrence of myocardial infarction, stroke, or CHD-related death (study definition of MACE).

### 2.3. Variables

#### 2.3.1. Race

Study participants were self-categorized as black or white in the ARIC [8] study.

#### 2.3.2. Major Adverse Cardiac Events (MACE)

MACE was defined as first occurrence of myocardial infarction, stroke, or CHD-related death from the baseline visit through 2004. This information was collected from study participants via annual phone interviews [9]. Self-reported diagnoses were verified by medical records for hospitalizations and outpatient cardiovascular events; next-of-kin interviews provided information on out-of-hospitals deaths which were subsequently reviewed and assigned a diagnosis [9].

#### 2.3.3. Covariates

Demographic characteristics (e.g., age, education, and marital status), health history, body mass index (BMI) kg/m^2^, and results of fasting laboratory assays were obtained from the baseline visit [8]. Family history included maternal and paternal CHD events. Current marital status (yes/no), high school graduate or more education (yes/no), current cigarette smoking (yes/no), and alcohol use (yes/no) were assessed at baseline. Measures of systolic and diastolic blood pressure were obtained and 12-hour fasting glucose, and cholesterol levels were assayed from blood samples at baseline. Participants taking antidiabetic medication or having fasting glucose ≥126 mg/dl were categorized as having diabetes mellitus. [10] Those taking cholesterol-lowering medication or having laboratory-assessed cholesterol >240 mmol/L were categorized as having high cholesterol. [11] Participants taking antihypertensive medications or having systolic pressure >140 mmHg or diastolic pressure >90 mmHg were categorized as hypertensive [12, 13]. Intake of current medications, including aspirin, antidiabetic medication, antihypertensive medication, and lipid lowering medication were determined by review of labelled containers [8] participants brought to the clinic visit.

#### 2.3.4. Genotyping

Whole genome genotyping was performed using the Affymetrix 6.0 array platform in this study population; [8] there were 82 *PON* SNPs (43 *PON1*, 32 *PON2*, and 7 *PON3*) available in the ARIC cohort which included (±) 20 kb window around each gene region. After excluding SNPs with minor allelic frequencies (MAF) less than 5%, 62 SNPs remained available for screening analysis. All SNPs were in Hardy–Weinberg equilibrium and had ancestry-specific allele frequencies similar to those reported in publicly available databases (https://www.ncbi.nlm.nih.gov/projects/gapsolr/facets.html). The 62 SNPs were screened for significant association with PAD in blacks and whites combined using a significance level of *p* < 0.001 (0.05/45) after Bonferroni correction for multiple comparisons confirmed 45 independent SNPs using the Nyholt method (https://neurogenetics.qimrberghofer.edu.au/matSpDlite/). Five principal component analysis covariates, obtained from PLINK [14] were used to adjust for residual population stratification. SNPs were coded as continuous variables for analysis using an additive model.

#### 2.3.5. Statistical Analysis

Descriptive statistics were calculated and reported as percentages for categorical variables and means (±standard deviations (SD)) for continuous data. Differences by race and MACE were analyzed using independent *t*-tests for continuous variables and chi-square tests for categorical variables. All covariates were noncollinear based on correlation coefficients of *r* < 0.30. Covariates with at least marginally significant differences by race and development of MACE, and known confounders were retained for further analysis. Statistical significance was defined as *p* < 0.05.

Forward stepwise Cox proportional hazards regression analysis was used to estimate the hazard ratio (HR) of having first occurrence MACE (myocardial infarction, stroke, or CHD-related death) by race, after adjustment for covariates. Kaplan–Meier analysis generated a survival curve depicting time to experience a first major adverse cardiac event for blacks and for whites. Covariates with a *p* value less than or equal to 0.05 were retained in the multivariable model. Assessment of the proportional hazards assumption (*p* < 0.05) was performed for all variables. Confounders, identified as variables yielding a 10% change in the estimated coefficient between full and reduced models, were retained in the final model. Model 1 examined the unadjusted association between race and MACE. Model 2 added age, gender, and BMI. Model 3 added cigarette and alcohol use. Model 4 added marital and educational status. Model 5 added aspirin use. Model 6 added high cholesterol, hypertension, and diabetes. Sensitivity analysis for familiar relatedness was conducted on all unrelated participants (*n* = 11,843) retaining the first enrolled member of each family when there was relatedness. None of the 62 *PON* SNPs screened met the significance criteria of *p* < 0.001 and were, therefore, not retained for further analysis. Interactions between race and covariates were evaluated with possible effect modification considered when *p* < 0.05.

## 3. Results

Table 1 shows baseline differences for blacks and whites in the ARIC cohort. Major adverse cardiac events (MACE) was found in 1,790 (14.0%) of the participants overall, with a higher proportion in blacks (17.7%) compared to whites (12.8%) (*p* < 0.0001). Blacks were younger (53.3 ± 5.8 vs. 54.3 ± 5.7 years, respectively, *p* < 0.0001), and had higher mean BMI (29.7 ± 6.1 vs. 27.0 ± 4.9 kg/m^2^, respectively, *p* < 0.0001) compared to whites. Compared to whites, black study participants were less likely to be men (*p* < 0.0001) and have paternal (*p* < 0.0001) or maternal (*p*=0.0002) family history of CVD. Blacks also had a lower proportion of current alcohol use (*p* < 0.0001) and fewer had attained a high school education (*p* < 0.0001), were married (*p* < 0.0001) or reported aspirin use (*p* < 0.0001) than whites. Rates of current smoking and diagnosed diabetes and hypertension were higher in blacks than whites (*p* < 0.0001). There was no difference in rates of diagnosed high cholesterol (*p*=0.11) between the races.

Comparisons of baseline characteristics by MACE in the ARIC cohort are shown in Table 2. Those with MACE were older (56.1 ± 5.5 vs. 53.8 ± 5.7 years, respectively, *p* < 0.0001) and had a higher average BMI (28.6 ± 5.3 vs. 27.5 ± 5.3 kg/m^2^, respectively, *p* < 0.0001). Compared to those without MACE, a greater proportion of participants with MACE reported being black, male (*p*‘*s* < 0.0001), more likely to have paternal (*p*=0.015) and maternal family history of CVD (*p* < 0.0001), more likely to be current cigarette users (*p* < 0.0001) and more likely to be diagnosed with hypertension, high cholesterol, and diabetes (*p*‘*s* < 0.0001). Additionally, fewer participants with MACE reported completing a high-school education (*p* < 0.0001), being currently married (*p*=0.0005), and currently using alcohol (*p* < 0.0001) compared to those without MACE. There was no difference between those with and without MACE for current aspirin use (*p*=0.21). None of the 62 *PON* SNPs in the ARIC population met the screening criterion for statistical significance (*p* < 0.001) and were, therefore, not retained for further analysis (Supplemental [Supplementary-material supplementary-material-1]).

**Figure 1:**
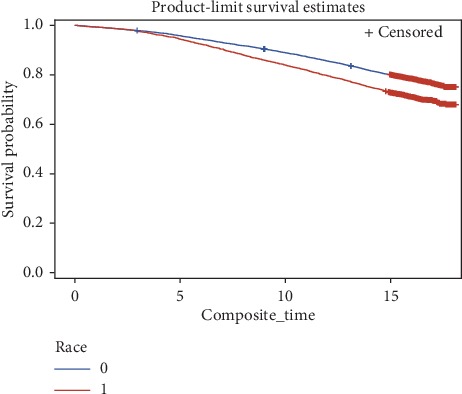
Kaplan–Meier curve for participants to reach first major adverse cardiac event (MACE); ARIC, 1987–1989 (*N* = 12770). White race (0), black race (1).

The Kaplan–Meier curve in Figure 1 graphically depicts the time to experience a first major adverse cardiac event is longer for whites compared to blacks. The association between race and MACE after forward step-wise adjustment for covariates in ARIC is shown in Table 3. Diabetes and hypertension were confounders and retained in the final model. The unadjusted hazard of MACE for blacks in comparison to whites was 1.46 (CI = 1.32,1.61, *p* < 0.0001, Model 1). After adjusting for age, gender, BMI, cigarette use, alcohol use, educational status and marital status, and current aspirin use, blacks had 1.24 times greater hazard of MACE than whites (OR = 1.24, 95% CI = 1.10, 1.39, Model 5). This association became nonsignificant in the subsequent Model 6 that further adjusted for high cholesterol, diabetes, and hypertension. Because of the potential for bias from familial relatedness, data were reanalyzed using only the 11,843 unrelated individuals (retaining the first enrolled member of each family when there was relatedness); comparable results were found in Models 1–6 with hazard ratios and confidence intervals similar to those observed in the full cohort.

Main effects for all variables in Model 6 are shown in Table 4. Age, male gender, cigarette use, high cholesterol, hypertension, diabetes (*p*‘*s* < 0.0001), BMI, and aspirin use (*p*‘*s* < 0.01) were all significantly and independently associated with higher hazard of MACE. High-school education attainment and alcohol use were significantly associated with lower hazard of MACE (*p* < 0.001). When effect modification between race and each covariate was tested, the only significant interaction was between race^*∗*^gender (*p*=0.03). When stratified by gender, race was not significant in either model; therefore, this interaction may be spurious and will be left out of the final model.

## 4. Discussion

Major adverse cardiac events (MACE) are the most commonly used composite end points in cardiovascular research to assess the safety and efficacy of treatment interventions for cardiovascular clinical events [15]. In this longitudinal analysis of 12,770 ARIC participants, blacks had 1.24 times greater hazard of MACE compared to whites after adjusting for age, gender, BMI, cigarette use, alcohol use, educational and marital status, and reported aspirin use. However, this association became nonsignificant after further adjustment for hypertension, diabetes, and high cholesterol. While this study did not find significant effect modification of *PON* SNPs on the association between race and MACE, to our knowledge, this is the first study to report results of such an evaluation.

In this study of men and women aged 45 years and older from the ARIC cohort, there was a higher prevalence of MACE in blacks than whites (17.7% vs. 12.8%). Previous studies have observed racial disparities in cardiovascular disease outcomes [16, 17]. A recent comprehensive narrative literature review summarized the well-established fact that blacks have higher rates of cardiovascular disease, including myocardial infarction and CHD-related death. [16] A comparative study by Feinstein et al., assessing racial differences in risk of first cardiovascular events, found that black men were more likely than white men to be diagnosed with CVD (HR 1.06; 95% CI 0.90, 1.26); however, black men were less likely than white men to have a first CVD event (HR 0.77; 95% CI 0.60, 1.00). [17] In contrast, our results suggested that race was not independently associated with MACE after adjustment for morbidities such as high cholesterol, hypertension, and diabetes, which are known CVD risk factors.

The lack of a significant association between race and MACE after adjustment for comorbidities in the present study suggests that differences in MACE may be influenced by racial disparities in risk factors and medical management rather than biological differences in the development of atherosclerosis. Attempting to understand racial disparities in CVD outcomes, Cram et al. reported that for coronary revascularization rates, blacks and Hispanics with similar insurance were significantly less likely to receive these procedures compared to whites [18]. Previous research reports that blacks present at a later clinical stage in the development of PAD than whites. Furthermore, diabetes and neuropathy, both more prevalent in blacks, affect the distal arteries and contribute to a PAD diagnosis [19]. Finally, it has been previously demonstrated that racial disparities exist in health care, with most blacks receiving lower quality than the majority of whites in the United States [20–24].

The most important consideration when selecting events to create a composite outcome is choosing events that participants perceive as being of equal importance and with similar impacts on health [4]. Here, we selected the first occurrence of myocardial infarction, stroke, and CHD-related death as the events to create the composite outcome. This was based on the composite scores most often used in previous studies and the individual outcomes available in the ARIC dataset. Few studies have been conducted using MACE as an endpoint for nontreatment or noninterventional studies in populations that are generalizable. For instance, a study conducted by Belonge et al. assessed the association of endurance exercise (which can exacerbate underlying cardiac issues) and major adverse cardiac events (death and hospitalization to coronary or myocardial infarction) and found the risk to be very low, occurring in 4 of 62,862 athletes [25].

The influence of genetic differences on the risk of MACE is relatively unknown. PON1 enzymes have anti-inflammatory and antioxidant properties and may protect against atherosclerosis. [26] The enzyme is expressed in the liver and delivered to multiple tissues not expressing the enzyme [26]. Decreased PON1 enzyme activity has been shown to increase inflammation in animal studies and increase oxidative stress among patients with atherosclerosis, diabetes. and/or hypercholesterolemia [26]. Numerous studies have been conducted assessing the association of the *PON* gene cluster and CVD; however, results have been inconclusive, suggesting more comprehensive multicentered studies are needed. [27] As discussed previously, the few previous studies assessing the effect of *PON* SNPs on the association between race and MACE composite outcome were among clinical (rather than population-based) samples undergoing cardiovascular treatment intervention [6, 7].

It is biologically plausible that there is an association between race and MACE after adjustment for biological risk factors. We may have failed to find this association in adjusted models because of the small sample of blacks relative to whites in this cohort. Future studies assessing the genetic differences and gene-environment interactions with respect to MACE need to be evaluated across diverse ethnic study populations with adequate sample sizes.

Several limitations and strengths of this study were considered. While MACE composite outcomes have been used since the mid 1990s, the research community continues to assess the validity and define the utility of this measure. Therefore, inherent weaknesses previously identified during the application of this composite measure must be acknowledged here. Misclassification due to self-identified race and ascertainment of other risk factors may contribute to residual confounding affecting the estimation of the association between race and MACE. This study also has several strengths including the use of data from a relatively large cohort of both black and white men and women who were enrolled using a standardized protocol. This study also adjusted for multiple behavioral and lifestyle covariates including educational status, which could contribute to differences in diagnosis and access to care and treatment. Finally, the effects of genetics as well as the interaction between race and MACE risk factors were examined.

## 5. Conclusions

While this study found an overall higher prevalence of MACE among black participants, race was not significantly associated with MACE after adjusting for comorbidities. This suggests the risk of MACE may be modified through medical management affecting disease outcomes. Additionally, racial disparities in health care may be the most significant contributing influence on the results observed here. Further research is warranted to better qualify the components included in the MACE composite outcome and to continue to assess the validity of this outcome measure representing cardiovascular disease risk.

## Figures and Tables

**Table 1:** Baseline characteristics by race^*∗*^; ARIC, 1987–1989 (*n* = 12770).

	Black (*n* = 3138)	White (*n* = 9632)	
	Mean (SD)	Mean (SD)	*p* value^a^
Age (yr)	53.3 (5.8)	54.3 (5.7)	<0.0001
BMI (kg/m^2^)	29.7 (6.1)	27.0 (4.9)	<0.0001
	N (%)	N (%)	
MACE	555 (17.7)	1235 (12.8)	<0.0001
Male	1175 (37.4)	4529 (47.0)	<0.0001
Family CVD history			
Paternal	561 (22.0)	3161 (35.5)	<0.0001
Maternal	428 (15.1)	1663 (18.0)	0.0002
Marital status	1852 (59.8)	8267 (87.1)	<0.0001
High school education	1873 (59.8)	8018 (83.4)	<0.0001
Current smoking status	919 (29.3)	2372 (24.6)	<0.0001
Current alcohol use	988 (31.8)	6269 (65.2)	<0.0001
Hypertension	1739 (55.7)	2587 (27.0)	<0.0001
High cholesterol	812 (27.2)	2475 (25.8)	0.1148
Diabetes	602 (19.7)	834 (8.7)	<0.0001
Aspirin	893 (28.9)	5033 (52.6)	<0.0001

^a^Race differences: comparisons performed with *t*-tests for continuous variables and chi-square tests for categorical variables.

**Table 2:** Baseline characteristics by major adverse cardiac events (MACE); ARIC (*n* = 12770).

	MACE^a^ (*n* = 1790)	No MACE (*n* = 10980)	
	Mean (SD)	Mean (SD)	*p* value^b^
Age (yr)	56.1 (5.5)	53.8 (5.7)	<0.0001
BMI (kg/m^2^)	28.6 (5.3)	27.5 (5.3)	<0.0001
	N (%)	N (%)	
Black race	555 (31.0)	2583 (23.5)	<0.0001
Male	1059 (59.2)	4645 (42.3)	<0.0001
Family CVD history			
Paternal	553 (35.1)	3169 (32.0)	0.0150
Maternal	355 (21.2)	1736 (16.7)	<0.0001
Marital status	1362 (77.3)	8757 (80.9)	0.0005
High school education	1186 (66.3)	8705 (79.4)	<0.0001
Current smoking status	640 (35.8)	2651 (24.2)	<0.0001
Current alcohol use	895 (50.3)	6362 (58.1)	<0.0001
Hypertension	958 (53.8)	3368 (30.8)	<0.0001
High cholesterol	596 (33.9)	2691 (24.8)	<0.0001
Diabetes	458 (25.8)	978 (9.0)	<0.0001
Aspirin	854 (48.2)	5072 (46.6)	0.2140

^a^MACE was defined as the first occurrence of myocardial infarction, stroke, or CHD-related death; ^b^comparisons performed with *t*-tests for continuous variables and chi-square tests for categorical variables.

**Table 3:** Association between race and major adverse cardiac events (MACE), adjusting for traditional risk factors for MACE and SNPs; results of Cox proportional hazard models with all participants and with data from only unrelated participants^c^; ARIC, 1987–1989.

All participants	N	HR (95% CI)	Variable (s) in model
Model 1	12770	1.46 (1.32,1.61)^a^	Race
Model 2	12751	1.55 (1.40,1.72)^a^	Model 1 + age, gender, and BMI
Model 3	12704	1.32 (1.18,1.47)^a^	Model 2 + alcohol use and cigarette use
Model 4	12512	1.19 (1.06,1.33)^b^	Model 3 + marital status and educational status
Model 5	12409	1.24 (1.10,1.39)^a^	Model 4 + current aspirin use
Model 6	12189	0.98 (0.87,1.11)	Model 5 + high cholesterol, diabetes, and hypertension
Only unrelated			

Participants	N	HR (95% CI)	Variable (s) in model

Model 1	11843	1.42 (1.31,1.54)^a^	Race
Model 2	11825	1.55 (1.43,1.69)^a^	Model 1 + age, gender, and BMI
Model 3	11781	1.33 (1.22,1.45)^a^	Model 2 + alcohol use and cigarette use
Model 4	11601	1.19 (1.08,1.30)^b^	Model 3 + marital status and educational status
Model 5	11505	1.22 (1.11,1.34)^a^	Model 4 + current aspirin use
Model 6	11299	1.04 (0.95,1.6)	Model 5 + high cholesterol, diabetes, and hypertension

Reference is the white race; ^*a*^*p* < 0.001; ^*b*^*p* < 0.05; ^c^in cases of familial relatedness, only data from the first enrolled individual were used.

**Table 4:** Adjusted independent associations of race and each covariate with major adverse cardiac events (MACE); results of Cox proportional hazard Model 6, ARIC, 1987–1989 (*n* = 12189).

	HR (95% CI)
Black race	0.98 (0.87,1.11)
Age (per 1 yr)	1.06 (1.05,1.07)^a^
Gender (male)	1.92 (1.68, 2.19)^a^
BMI (kg/m^2^)	1.02 (1.01,1.03)^b^
Marital status (yes)	0.89 (0.78, 1.00)
Educational status (yes)	0.78 (0.70,0.87)^a^
Cigarette use (yes)	1.91 (1.67,2.19)^a^
Alcohol use (yes)	0.79 (0.71,0.87)^a^
Aspirin use (yes)	1.11 (1.00,1.23)^b^
High cholesterol (yes)	1.36 (1.22,1.50)^a^
Diabetes (yes)	2.28 (2.03,2.56)^a^
Hypertension (yes)	1.15 (1.05,1.27)^a^

Reference is the white race; Model adjusted for race, age, gender, BMI, cigarette use, alcohol use, marital status, educational status, aspirin use, high cholesterol, diabetes, and hypertension; ^*a*^*p* < 0.001; ^*b*^*p* < 0.01; ^c^in cases of familial relatedness, only data from the first enrolled individual were used.

## Data Availability

The Atherosclerosis Risk in Communities Study (ARIC) dbGaP data used to support the findings of this study are included within the article.

## Supplementary Materials

Supplementary Materials Supplemental Table A: association of SNP with major adverse cardiac events (MACE) by race; screening results of univariate logistic regression, ARIC, 1987–1989.
