# Supplementary material for: Association of Race and Major Adverse Cardiac Events (MACE): The Atherosclerosis Risk in Communities (ARIC) Cohort
Source: J Aging Res. 2020 Mar 21;2020:7417242. doi: 10.1155/2020/7417242 (PMC7114773; doi:10.1155/2020/7417242)
Supplement: Supplementary Materials — Supplemental Table A: association of SNP with major adverse cardiac events (MACE) by race; screening results of univariate logistic regression, ARIC, 1987–1989. [file 7417242.f1.zip › 7417242.f1/7417242.v2 (5) 24.pdf]

Supplemental Table A. Association of SNP with major adverse cardiac events (MACE) by race; screening results of univariate logistic regression, ARIC, 1987-1989

	Black	White
	p-value	p-value
PON1 SNPs		
rs2057681	0.5912	0.0446‡
rs3917527	0.1525	0.0039‡
rs2301711	0.9103	0.0158‡
rs2299260	0.4784	0.6721
rs2299261	0.6599	0.5739
rs854568	0.6431	0.4192
rs13223537	0.3682	0.6726
rs705378	0.7088	0.5253
rs854569	0.1142	0.4523
rs17166829	0.0330‡	0.9589
rs3917538	0.2786	0.5578
rs3917521	0.3223	0.6179
rs854565	0.9087	0.0240‡
rs854566	0.9492	0.2523
rs2237583	0.3767	0.1864
rs854572	0.1852	0.9575
rs3917541	0.3658	0.0049‡
rs3917551	0.2052	0.0041‡
rs3917550	0.9750	0.4234
rs2074354	0.2849	0.8526
rs3917490	0.6963	0.7713
rs2299262	0.2017	0.4169
rs854571	0.0245‡	0.6734
rs13236941	0.0370‡	0.7080
rs2272365	0.4625	0.8396
rs705382	0.2693	0.9339
rs2269829	0.5676	0.0483‡
rs2299257	0.8453	0.0799
PON2 SNPs		
rs2299267	0.9991	0.9154
rs43037	0.0424‡	0.7042
rs7778623	0.9760	0.6162
rs43052	0.4252	0.6123
rs4729190	0.8554	0.3005
rs1557782	0.9967	0.5502
rs43063	0.6183	0.6388
rs6958904	0.8288	0.7846
rs2299263	0.6084	0.7650
rs7785039	0.4878	0.9684
