# Supplementary material for: Association of Race and Major Adverse Cardiac Events (MACE): The Atherosclerosis Risk in Communities (ARIC) Cohort
Source: J Aging Res. 2020 Mar 21;2020:7417242. doi: 10.1155/2020/7417242 (PMC7114773; doi:10.1155/2020/7417242)
Supplement: Supplementary Materials — Supplemental Table A: association of SNP with major adverse cardiac events (MACE) by race; screening results of univariate logistic regression, ARIC, 1987–1989. [file 7417242.f1.zip › 7417242.f1/7417242.v2 (5) 25.pdf]

rs3757707	0.1712	0.7622
rs43061	0.6193	0.4339
rs43065	0.1491	0.5700
rs2374993	0.8612	0.7983
rs10241004	0.8500	0.8825
rs10261470	0.6424	0.6136
rs10953151	0.4355	0.8897
rs6973380	0.8062	0.7005
rs10487133	0.0163‡	0.9451
rs7493	0.8390	0.7165
rs12534203	0.4505	0.9360
rs10953149	0.4896	0.9430
rs12535571	0.8993	0.9899
rs1639	0.3253	0.5626
rs43044	0.6536	0.2165
rs6950550	0.6212	0.4517
rs12530498	0.5058	0.5507
rs43048	0.8311	0.1943
rs7802018	0.6417	0.7300
PON3 SNPs		
rs468	0.2708	0.7405
rs1053275	0.7518	0.7637
rs11768074	0.8650	0.8064
rs9641162	0.0935	0.6589
rs10953143	0.1210	0.8951

References: *p<0.001; ‡p<0.05
